# Prospective Study of the Evolution of Blood Lymphoid Immune Parameters during Dacarbazine Chemotherapy in Metastatic and Locally Advanced Melanoma Patients

**DOI:** 10.1371/journal.pone.0105907

**Published:** 2014-08-29

**Authors:** Grégoire Mignot, Alice Hervieu, Pierre Vabres, Sophie Dalac, Geraldine Jeudy, Blandine Bel, Lionel Apetoh, François Ghiringhelli

**Affiliations:** 1 INSERM, UMR866, Dijon, France; 2 Faculté de Médecine, Université de Bourgogne, Dijon, France; 3 Service de Dermatologie, Centre Hospitalier Universitaire le Bocage, Dijon, France; 4 Oncologie médicale, Centre Georges François Leclerc, Dijon, France; University of Queensland Diamantina Institute, Australia

## Abstract

**Background:**

The importance of immune responses in the control of melanoma growth is well known. However, the implication of these antitumor immune responses in the efficacy of dacarbazine, a cytotoxic drug classically used in the treatment of melanoma, remains poorly understood in humans.

**Methods:**

In this prospective observational study, we performed an immunomonitoring of eleven metastatic or locally advanced patients treated with dacarbazine as a first line of treatment. We assessed by flow cytometry lymphoid populations and their activation state; we also isolated NK cells to perform in vitro cytotoxicity tests.

**Results:**

We found that chemotherapy induces lymphopenia and that a significantly higher numbers of naïve CD4^+^ T cells and lower proportion of Treg before chemotherapy are associated with disease control after dacarbazine treatment. Interestingly, NK cell cytotoxicity against dacarbazine-pretreated melanoma cells is only observed in NK cells from patients who achieved disease control.

**Conclusion:**

Together, our data pinpoint that some immune factors could help to predict the response of melanoma patients to dacarbazine. Future larger scale studies are warranted to test their validity as prediction markers.

## Introduction

In the last few years, there have been many considerable changes in the treatment of metastatic melanoma, with the development of BRAF and MEK tyrosine kinase inhibitors and monoclonal antibody immunotherapies, such as anti CTLA-4 and anti PD-1, which have proved efficacy both in term of clinical response and overall survival. However, in metastatic melanoma patients who presented side effects, no indications or failure of targeted therapies or immunotherapy, “classical” cytotoxic chemotherapy is still used, namely dacarbazine (DTIC).

It has been shown that some cytotoxic drugs could affect the immune system and the antitumor immune response. The first mechanism is directly related to the cytotoxic property of these agents on cancer cells. The seminal discovery was that cancer death induced by some chemotherapies could prime CD8^+^ T cell antitumor immune response. This phenomenon is an essential contributor to the antitumor effect of some major anticancer drugs such as anthracyclines and oxaliplatin both in mice and humans [1]–[4]. The second phenomenon involves the capacity of some anticancer agents to selectively kill or affect the biology of some immune cells. Anticancer drugs can eliminate immunosuppressive cells and enhance antitumor immune responses [5]–[7] or mitigate cytotoxic antitumor immunity by inducing some immunosuppressive mechanisms [8]. In a recent work, using a mouse melanoma model B16F10, we identified DTIC immunological effect. While DTIC did not directly affect immune cells in this mouse model, we observed that DTIC triggered the expression of NKG2D ligands on tumor cells that led to activation of natural killer (NK) cells, interferon (IFN)γ secretion, then activation of cytotoxic T cells [9], [10]. We also observed that *in vitro* DTIC treatment enhanced NKG2D ligand expression on human melanoma cell lines. We thus formulated the hypothesis that for some patients, DTIC may also enhance NK cell toxicity toward melanoma cells and that this could be related to the clinical response to DTIC.

To address this question, we performed immunomonitoring of lymphoid subpopulations of patients with metastatic melanoma before and after a first cycle of DTIC treatment.

## Material & Methods

### Patients

To monitor immune markers that could potentially be implicated in melanoma as prognosis and/or prediction for treatment response, we immunomonitored patients from the university Hospital of Dijon (France), bearing unresectable or metastatic melanoma. All patients gave their written informed consent and were treated with the cytotoxic chemotherapy DTIC at 1 g/m^2^ every four weeks. The response rate was evaluated one month after the third cycle of treatment by CT scan, according to RECIST criteria 1.1. This trial received approbation of the ethics committee of the Hospital of Dijon under the number 2010/55 eudract n: 2010-02358-34 and was conducted following the Declaration of Helsinki's protocol. Patient whose death was related to cancer are counted as “dead”, the others are censored after their last sign-of-life.

### Blood sample preparation

Blood samples were obtained from patients before the first and second cycle of chemotherapy. Blood was collected in vacutainer tubes, with EDTA for determination of total leukocytes, lymphocytes and granulocytes concentration or with citrate for immunomonitoring. Citrated samples were diluted 1∶1 with RPMI1640 (Lonza) and centrifugated on a cushion of lymphocyte separation medium (Eurobio). Peripheral blood mononuclear cells (PBMC) were collected and washed once with phosphate buffered saline (PBS) 5% bovine serumalbumin (BSA). 5% of the pellet was taken for further polymerase chain reaction (PCR) analysis, 10% for flow cytometry analysis and the remaining cells underwent NK cell separation with an NK cell isolation kit (Miltenyi Biotech), followed by separation on AutoMACS (Miltenyi).

### Cytotoxicity assay

The MelC human melanoma cell line was a kind gift from Dr Anne Caignard. MelC cells, previously treated with PBS or DTIC (0.4 g/L) were incubated overnight either alone or with freshly purified NK cells from patients. Tumor cells were harvested and death was assayed using DAPI (4′,6′-diamidino-2-phénylindole) staining (Sigma Aldrich) by flow cytometry. Tumor cells were identified by their characteristic size and granularity, and cells positives for DAPI staining were considered dead.

### PCR analysis

Total RNA from PBMC was extracted using TRIZOL (Invitrogen) as recommended by the manufacturer. The RNAs were rehydrated and measured with a NanoDrop 1000 (ThermoScientific). Reverse transcription was performed using the kit "M-MLV Reverse Transcriptase" (Invitrogen) as recommended by the manufacturer using a Biometra thermocycler. The quantitative PCR reaction was performed using the kit “SYBR Green PCR Master Mix” (Applied Biosystems) according to manufacturer's recommendations, on an Applied 7500 device (Applied Biosystems). Relative mRNA levels were determined using the difference in cycle threshold (ΔCt) method. Values were expressed relative to *ACTB*. We detected *ACTB* (with primers GATCATTGCTCCTCCTGAGC and TGCGCAAGTTAGGTTTTGTC), *EOMES* (CAGCACCACCTCTACGAACA and CGCCACCAAACTGAGTGAT), *TBX21* (AACATCCTGTAGTGGCTGGTG and CCACCTGTTGTGGTCCAAGT) and *RORC* (AAGCAGGAGCAATGGAAGTG and GCAATCTCATCCTCGGAAAA).

### Flow cytometry

Blood counts (leukocytes, granulocytes, lymphocytes and monocyte counts) were performed on the Xn2000 (Sysmex) device, which is the device used for clinical blood count in Dijon's hospital routine care. Fluorescent data were produced using a LSRII cytometer (BD Pharmingen). PBMC were stained in PBS/5%BSA with antibodies indicated in Table 1. All antibodies were purchased from Biolegend (Ozyme) and used according to the manufacturer's protocol. Analyses were done with FlowJo software (Tristar). Events were considered positive if their fluoresence was higher than matched- isotype control staining peak fluorescence. The gating strategy is summarized in Fig. S1 & S2.

**Table 1:** Antibodies for flow cytometry analysis.

Antigen	Clone	Panel 1	Panel 2	Panel 3
CD3	UCHT1	PE	PE/Cy7	PE
CD4	RPA-T4	PerCP		PerCP
CD8	HIT8a	PE/Cy7		
CD16	3G8		Alexa fluor 700	
CD45RA	HI100	Brillant Violet 570		
CD56	MEM-188		PE	
CD69	FN50	Alexa Fluor 647	Alexa Fluor 488	
FOXP3	206D			Alexa Fluor 647

PerCP: Peridinin chlorophyll; PE: Phycoerythrin; Cy7: Cyanine-7.

### Statistical analysis

We used Mann-Whitney's test for nonparametric means comparison. Student's t test was used when variance were tested comparable by Fisher-Snedecor's test. Paired tests were used when applicable. Linear correlations were assessed using the Pearson correlation coefficient. All tests were performed and graphed with GraphPad Prism 6 software. All p values were two-tailed. A p value inferior to 0.05 was considered statistically significant for all experiments and is indicated with *.

## Results

### Patients

Eleven patients were included in this prospective observational trial. Patients' clinical characteristics are described in Table 2. In our cohort, one patient exhibited partial response, two showed disease stabilization and eight had progressive disease following DTIC treatment. Patients that showed partial response or disease stabilization after DTIC were grouped as “disease control”. So, in our cohort, there were 3 (27%) patients with disease control and 8 (73%) with progressive disease, which is consistent with the published response rates to DTIC in the literature [11], [12]. Survival analysis (Fig. S3) underscores that patients with progressive disease have a shorter survival than those with disease control (median survival 7 vs 19.5 months, respectively, Mantel-Cox’test p = 0.0379).

**Table 2:** Patients' clinical characteristics.

Patient n°	Age	Sex	Histological type	Breslow score (mm)	Stade	Metastasis	Response	Survival (month)
1	85	Female	Desmoplastic	4	IIIC	Skin, lymph node	PR	18†
2	87	Female	Mucosal	N/A	II (*)		PD	6†
3	88	Female	Lentigo maligna	3,1	IV M1b	Lung, Lymph node	SD	21No treatment
4	77	Male	Lentigo maligna	11	IV M1b	Lung, skin	PD	13†
5	66	Female	N/A	2,6	IIIC	Skin, lymph node	PD	1Ipilimumab
6	95	Male	Superficial-spreading	3,5	IV M1b	Lung, skin, lymph node	PD	3†
7	62	Male	Superficial-spreading	1,1	IV M1c	Lung, lymph node, liver, bone, thyroide	PD	8†
8	69	Male	N/A	3,5	IV M1b	Lung	PD	12Ipilimumab
9	44	Female	Superficial-spreading	1,6	IV M1c	Lung, lymph node, liver, brain	PD	3†
10	74	Female	Acral-lentiginous	2,4	IIIC	Skin	SD	9No treatment
11	72	Female	Superficial-spreading	1,31	IV M1a	Skin, lymph node	PD	9†

PD: progressive disease.

PR: partial response.

SD: stable disease.

† : dead.

(*): Patient n°2 was inoperable, due to initial extension of its nasal tumor.

### Blood count

**Figure 1:**
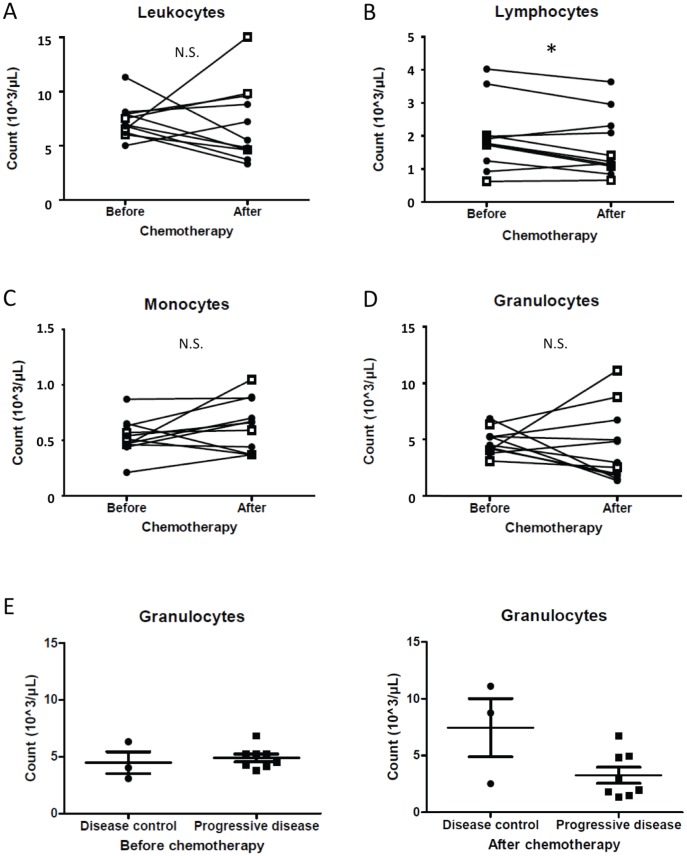
Chemotherapy causes changes in blood cells. We retrieved clinical data about leukocytes (A), lymphocytes (B), monocytes (C) and granulocytes (D) before the first (“Before”) and second (“After”) DTIC treatment. Black filled diamonds represent data from patients with progressive disease and white squares represent data from patients with disease control. For panel E, we compared granulocytes counts between patient groups before the first and second treatment (left and right panel, respectively).

A total blood count was performed before the first and second cycle of chemotherapy. One injection of DTIC induced no change in the total number of leucocytes (Fig. 1A). When looking at leukocyte types, we observed a drop in total lymphocyte count (Fig. 1B, p = 0.0439), whereas no significant modifications were observed for monocyte (Fig. 1C) or granulocyte counts (Fig. 1D). Neither lymphocyte nor monocyte counts before or after chemotherapy were associated with disease control (not shown). Surprisingly, patients with disease control showed a tendency of higher granulocyte counts after treatment compared to the progressive disease group (Fig. 1E).

### T cells

**Figure 2:**
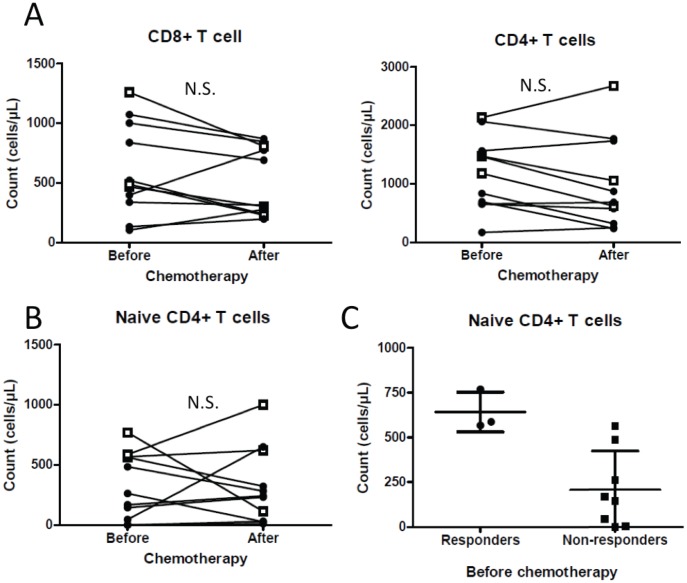
Treatment impacts T cell sub-populations. Panel A shows CD8^+^ (left panel) and CD4^+^ (right panel) T cell counts before and after chemotherapy. B and C: Naïve CD4^+^ T cell counts before and after chemotherapy (B) and between patient groups (C). Black filled diamonds represent data from patients with progressive disease and white squares represent data from patients with disease control.

We did not observe differences in total number of CD4^+^ or CD8^+^ T cells before and after chemotherapy (Fig. 2A), although a tendency could be observed for both, accounting for the diminution of total lymphocyte count (Fig. 1B). When looking at memory and naïve subpopulations, we observed that chemotherapy did not significantly affect the number of naïve CD4^+^ T cells (Fig. 2B) or naïve CD8^+^ T cells (not shown). However, high numbers of naïve (CD45RA^+^) CD4^+^ T cells before chemotherapy are seen in patients with controlled disease (Fig. 2C). Neither the numbers of CD4^+^ or CD8^+^ T cells after one cycle of chemotherapy, nor naïve CD8^+^ T cell number are correlated with clinical outcome (Not shown).

### T cell polarization

**Figure 3:**
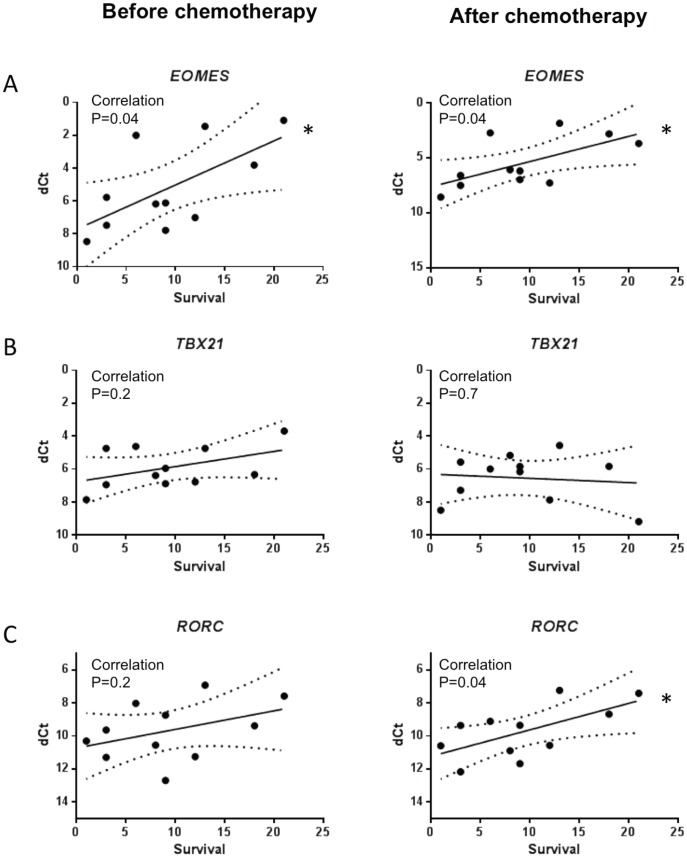
Correlation between T cell transcription factors and survival. We performed RT-qPCR on RNA from PBMC. Figure depicts relation between survival (in months) and expression level of indicated genes: *EOMES* (A), *TBX21* (B), *RORC* (C).

The polarization state of T cells was determined using the monitoring of master controller transcription factors using RT-qPCR. There was a strong correlation between expression of *EOMES*, coding for eomesodermin, before and after treatment and survival (Fig. 3A), thus suggesting that CD8^+^ or NK cells anti-tumor cytotoxic responses would be associated with longer survival. We did not find any correlation between the expression of *TBX21*, a gene coding for T-bet, before and after chemotherapy and overall survival (Fig. 3B). Conversely, after chemotherapy, the Th17 transcription factor, *RORC*, was associated with longer survival (Fig. 3C).

### Regulatory T cells

**Figure 4:**
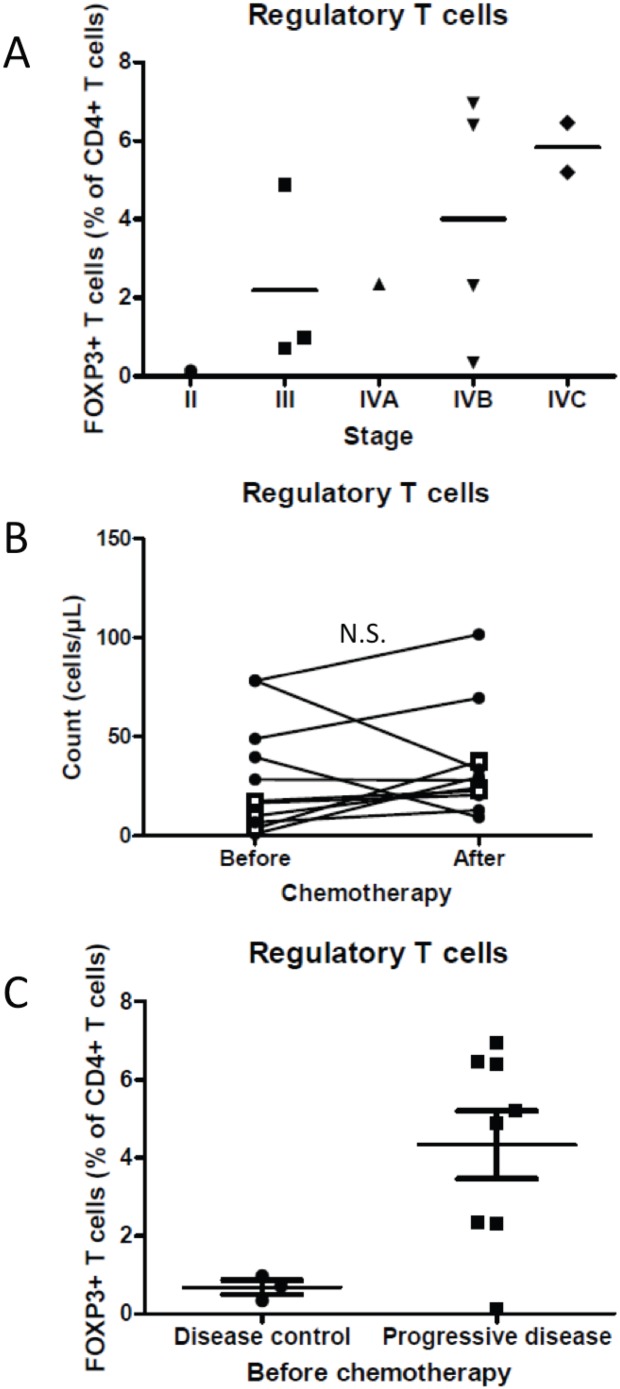
Regulatory T cells are associated with tumor grade and response to chemotherapy. A: The proportion of FOXP3^+^ amongst CD4^+^ T cells (Treg) is depicted in function of tumor grade of the corresponding patient. B: Treg numbers are depicted before and after treatment with DTIC. Black filled diamonds represent data from patients with progressive disease and white squares represent data from patients with disease control. C: Proportion of Treg amongst CD4^+^ T cells.

We evaluated the proportion of FOXP3 positive cells amongst the CD3^+^CD4^+^ double-positive lymphocyte cells. There was a tendency for the proportion of FOXP3 expression to positively correlate with tumor stage (Fig. 4A). We noted that chemotherapy did not significantly affect FOXP3^+^ cell numbers (Fig. 4B). However, we observed that, before chemotherapy, patients with disease control had fewer FOXP3^+^ than those with progressive disease (Fig. 4C).

### NK

**Figure 5:**
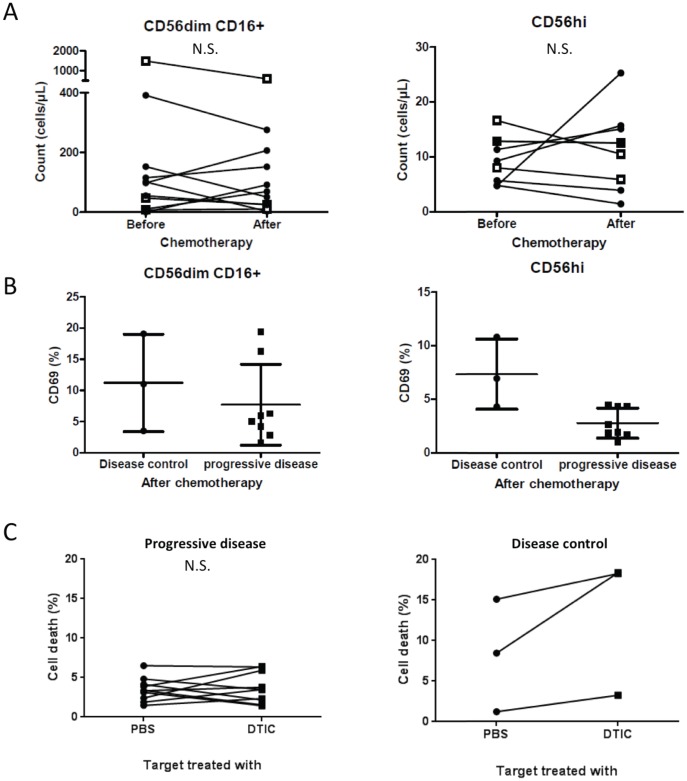
NK cell activity is related to chemotherapy response. A: CD56^dim^CD16^+^ (left panel) cells and CD56^hi^CD16^−^ (right panel) NK cells counts. Black filled diamonds represent data from patients with progressive disease and white squares represent data from patients with disease control. B: CD69 expression by CD56^dim^CD16^+^ (left panel) cells and CD56^hi^CD16^−^ NK cells (right panel). C: Death of MelC cells pretreated with PBS or DTIC, and cocultured with NK cells isolated from patients.

Treatment had no significant effect on total number of either CD56^hi^ or CD56^dim^CD16^+^NK cell subsets (Fig. 5A). We also did not detect significant modifications of the activation state of NK cell subsets after chemotherapy using CD69 labeling (Fig. S4). Furthermore, we did not see any association between CD56^dim^CD16^+^ NK cell activation and response. However, we observed a higher activation of CD56^hi^ NK cells in patients with disease control after chemotherapy (Fig. 5B) thus suggesting that DTIC may help activation of CD56^hi^ NK cells in these patients.

We previously demonstrated that, in a mouse model, DTIC induces the expression of NK cell activating ligands on the surface of melanoma cells. This causes a better detection and lysis of DTIC-treated targets by NK cells [10]. To validate this observation in a human context, we tested in this cohort the cytotoxic properties of purified NK cells from patients before chemotherapy, against a human melanoma cell line *ex vivo* pretreated with DTIC at a dosage that triggers expression of NKG2D ligand [10]. We observed that NK purified from patients with progressive disease are unable to lyse either DTIC-treated targets or PBS-treated targets [13] (Fig. 5C, left panel), with a cytotoxicity -if any- comparable to the background mortality, i.e. the death of target cells cultured without NK cells, that is 6+/−1% (IC95[3.7–8.2]) and 5+/−1% (IC95[2.1–8.2]) for PBS- and DTIC-treated MelC cell, respectively. In contrast, NK cells from patients with disease control seem to have better capacity to kill pretreated DTIC target cells (Fig. 5C, right panel).

## Discussion

To our knowledge, this study is the first that performs a systematic study of the effect of alkylating chemotherapy on all blood lymphoid cell subsets in melanoma patients. We observed that chemotherapy modestly but significantly affects the total number of lymphocytes, which is reduced after chemotherapy. This observation could be linked to the toxicity of this anticancer drug. However chemotherapy did not affect the number of CD8^+^ or CD4^+^ T cells or Treg or NK cells thus underscoring that such treatment is not a major immunosuppressive treatment.

High expression of *EOMES* gene, encoding eomesodermin is also correlated with longer survival. Eomesodermin has been described as a master regulator of cytotoxic CD8^+^ T cells [14], and can also be expressed by NK cells in mice [15]. This suggest that the intrinsic presence of a cytotoxic cellular response could be important for DTIC efficacy, thus corroborating our previous mouse findings that DTIC antitumor effects are dependent on CD8 T cells and NK cells [10].

After treatment, high expression of *RORC*, coding for RORγt, a transcription factor responsible for CD4^+^ Th17 differentiation, correlates with better survival. Th17 cell induction has already been described with various melanoma treatments, such as vaccination [16], anti-PD-1 [17], anti-CTLA4 [18], as well as other anticancer drugs such as cyclophosphamide [19]. Furthermore, it has been described that Th17 cells can drive antigen-dependent tumor shrinkage [20], [21]. We hypothesize that Th17 cell induction after treatment could be a surrogate marker of efficacy. This event could be related to the association of high granulocyte counts in responder patients after treatment, because Th17 cells are well known to be important in the activation and recruitment of neutrophils.

CD4^+^ T cell subsets seem to be associated with clinical outcome. In particular, the presence of higher number of naïve CD4^+^ T cells at baseline is associated with better response to DTIC. In patients with low numbers of naïve CD4^+^ T cells, we suspect that there is an increase in the number of memory and exhausted lymphocytes. Indeed, in chronic inflammatory situations such as cancer, the presence of chronic antigen stimulation leads to an exhausted or senescent memory phenotype of CD4^+^ T cells. These cells are known to dampen anti-tumor immune response efficacy [22]. Such cells are known to accumulate in peripheral blood of cancer patients [23]. It is possible that the high number of exhausted lymphocytes could impair chemotherapy associated antitumor immune response, thus rendering such drug less effective. In addition, our study corroborates the previous finding that higher Treg rate is associated with advanced clinical stage [24]. Moreover, higher Treg number at baseline is associated with absence of response to DTIC. Such data suggest that high tumor burden and/or immunosuppressive state could impair antitumor efficacy of DTIC probably by inhibiting drug related antitumor immune response.

The major finding of this study is the relationship between NK cell activity and response to DTIC. We analyzed the two subsets of peripheral blood NK cells, that are described as functionnaly different[25]: CD56^hi^ NK cells can secrete high amounts of cytokines [26], and CD56^l^°CD16^+^ are perceived as cytotoxic [27]. Our results show that, while DTIC did not affect the total number of NK cells, CD56^hi^ NK cells from patients with disease control have a higher activation status than the patients with progressive disease. We previously showed that DTIC can enhance NKG2D ligand expression by melanoma cells [10]. In a mouse model, we showed that this causes IFNγ secretion by NK cells, followed by major histocompatibility complex expression by tumor cells, hence better recognition by T cells. We selected here a melanoma cell line in which DTIC *ex vivo* treatment induced up regulation of NKG2D ligands. We and others already described how these cells are killed by NK cells thanks to NKG2D-recognition [9], [10], [28]. Using this setting, we could see here that NK cells from responder patients seem to have a stronger capacity to kill DTIC- pretreated cancer cells than NK cells from patients with progressive disease. This is coherent with the published literature, as Anne Caignard's group described how NK cell phenotypes and functions vary according to tumor stage and treatment [28], [29]. Indeed, they showed that NK cell receptor NKP46 expression by NK cells seems diminished in patient bearing metastatic melanoma, and that chemotherapy could increase expression of NKP46 by NK cells, but IFNγ secretion and degranulation are decreased after chemotherapy in ex-vivo assays, due to up-regulation of inhibiting factor NKG2A [28], [29]. They also showed no variation of NKG2D expression by NK cells after chemotherapy or according to tumor stage. Our data support that alkylating chemotherapy such as DTIC is only effective in patients with functional NK cells and support that either some patients have intrinsic more proficient NK cells or that the tumor drives an immunosuppressive state, which blunts NK cell function. The second hypothesis is more probable because we described that pretreatment of melanoma cell line with DTIC enhanced the cytotoxicity of NK cells from healthy volunteers [10] and that high number of Treg before chemotherapy is associated with absence of response to DTIC.

Taken together, our study underscores some immune properties of DTIC and gives some preliminary data to isolate predictive factors of response. We believe that the two most promising factors are the importance of naïve CD4 T cell number and NK cell activity to predict response to chemotherapy. First, our results show that high Treg number and low number of naïve CD4^+^ T cells are associated with disease progression after chemotherapy. These patients are presumably those with exhausted memory cells and may greatly benefit from immune-based treatments, such as anti-CTLA4 or anti-PD1 antibody, because these two treatments aim to reverse immune inhibition and exhaustion. Furthermore, patients having highly cytotoxic NK cells could be better candidates to DTIC-based treatment, as they may benefit the most from its immune effects, enhancing NK- and CD8^+^T cell based cytotoxicity. Larger studies are warranted to validate these results.

## Supporting Information

Figure S1 **Flow cytometry strategy.** We present here the gating strategy for flow fluorocytometry analysis. We present two examples of NK cell CD69 labelling.
(EPS)

Figure S2 **Flow cytometry strategy for FOXP3 analysis.** We present gating strategy for FOXP3+ analysis by flow fluorocytometry. We show two examples of FOXP3 labelling.
(EPS)

Figure S3 **Survival curves.** Comparison of survival curves of patient with progressive disease or disease control.
(EPS)

Figure S4 **Activation of NK cells is not related to chemotherapy.** We compared NK cell activation before and after chemotherapy. We used CD69 labelling by flow cytometry to assess activation of CD56^hi^ (A) and CD56^dim^CD16^+^ NK cells (B).
(EPS)
